# Exploring Patient Needs and Designing Concepts for Digitally Supported Health Solutions in Managing Type 2 Diabetes: Cocreation Study

**DOI:** 10.2196/49738

**Published:** 2023-08-25

**Authors:** Dan Roland Persson, Katiarina Zhukouskaya, Anne-Marie Karin Wegener, Lene Kølle Jørgensen, Jakob Eyvind Bardram, Per Bækgaard

**Affiliations:** 1 Department of Applied Mathematics and Computer Science Technical University of Denmark Kgs. Lyngby Denmark; 2 Danish Diabetes Association Glostrup Denmark; 3 Department of Health Technology Technical University of Denmark Kgs. Lyngby Denmark

**Keywords:** cocreation, co-creation, participatory design, diabetes, type 2 diabetes mellitus, T2DM, chronic illness, personalized self-care, mobile health, mHealth, eHealth

## Abstract

**Background:**

Self-management of the progressive disease type 2 diabetes mellitus (T2DM) becomes part of the daily life of patients starting from the time of diagnosis. However, despite the availability of technical innovations, the uptake of digital solutions remains low. One reason that has been reported is that digital solutions often focus purely on clinical factors that may not align with the patient’s perspective.

**Objective:**

The aim of this study was to develop digital solutions that address the needs of patients with T2DM, designed from the user’s perspective. The goal was to address the patients’ expressed real-world needs by having the users themselves choose the scope and format of the solutions.

**Methods:**

Using participatory methods, we conducted 3 cocreation workshops in collaboration with the Danish Diabetes Association, with 20 persons with T2DM and 11 stakeholders across workshops: user experience designers, researchers, and diabetes experts including a diabetes nurse. The overall structure of the 3 workshops was aligned with the 4 phases of the double diamond: initially discovering and mapping out key experienced issues, followed by a workshop on thematic mapping and definition of key concepts, and succeeded by an exploration and development of 2 prototypes. Subsequently, high-fidelity interactive prototypes were refined as part of the delivery phase, in which 7 formative usability tests were conducted.

**Results:**

The workshops mapped experiential topics over time from prediagnosis to the current state, resulting in a detailed exploration and understanding of 6 themes related to and based on the experiences of patients with T2DM: diabetes care, diabetes knowledge, glucose monitoring, diet, physical activity, and social aspects of diabetes. Two prototypes were developed by the participants to address some of their expressed needs over time related to the 6 themes: an activity-based continuous glucose monitoring app and a web-based guide to diabetes. Both prototypes emphasize periods of structured self-measurements of blood glucose to support evolving needs for self-exploration through distinct phases of learning, active use, and supporting use. Periods of low or intermittent use may thus not reflect a failure of design in a traditional sense but rather be a sign of evolving needs over time.

**Conclusions:**

Our results indicate that the needs of patients with T2DM differ between individuals and change over time. As a result, the suggested digitally supported empowering health prototypes can be personalized to support self-exploration, individual preference in long-term management, and changing needs over time. Despite individuals experiencing different journeys with diabetes, users perceive the self-measurement of blood glucose as a universally useful tool to empower everyday decision-making.

## Introduction

### Background

Type 2 diabetes mellitus (T2DM) is a progressive, chronic metabolic disorder characterized by elevated blood glucose levels. Uncontrolled diabetes can lead to severe complications including cardiovascular disease, neuropathy, nephropathy, and retinopathy. Effective management is crucial for improving patient outcomes and reducing the burden of diabetes-related complications.

The management of T2DM involves lowering the average blood glucose level as measured from hemoglobin A_1c_ (HbA_1c_), through medication, diet, and exercise. Controlling blood pressure and blood lipids are also vital but will not be addressed in this paper. An important component of the HbA_1c_ level is the postprandial glucose concentration 1.5 to 2 hours after a meal. This concentration is influenced by the composition and size of the meal and by the amount of exercise and can be measured using, for example, the structured self-monitoring of blood glucose (SMBG) or continuous glucose monitoring (CGM).

According to the World Health Organization, 1 in 11 people have diabetes, corresponding to 442 million globally [1], with estimates suggesting that as many as 700 million people will be affected by 2045 and another 548 million people will be in the prestages of diabetes [2]. Many patients with T2DM are left on their own most of the time with very limited contact with health care professionals. Barnes et al [3] and Chen et al [4] showed that most patients with T2DM spend less than 5 hours annually with specialists, leaving a tremendous burden on the patients with T2DM in the form of self-care and self-management.

Supporting patients with T2DM to better manage their disease themselves has been suggested as a promising way to improve patients’ health and quality of life and reduce the chronic disease management burden [5-7]. T2DM entails a significant number of challenges for the individual and can be largely asymptomatic until the onset of diabetes complications, making it difficult for people to tell how they are doing unless actively monitored [8]. Despite many innovations, technical breakthroughs, and research, diabetes control in the United States has deteriorated in the recent decade [9].

Mobile health (mHealth) technology has received significant attention in recent years because such solutions are both highly scalable and cost-effective [10], with some reviews cautiously suggesting a moderately sized effect on improving disease outcomes relative to traditional treatments [11,12]. Nevertheless, high attrition rates and lowered long-term engagement continue to be challenges. Meyerowitz-Katz et al [13] reported 43% dropout rates in mHealth studies on chronic diseases, with a 49% rate across observational studies, noting that true attrition rates may be higher than those reported, given unclear definitions, and more so outside controlled study settings [13,14].

This indicates a lack of user acceptance, possibly caused by a failure to address specific and evolving user needs [15]. Although the exact mechanisms behind attrition are not understood, one possible factor could be that “patients’ experiences and needs might not always align with clinical judgment” [16]. Although some suggested apps address user engagement to improve long-term engagement [17,18], they may still need to address user preferences [19] and the need for self-exploration [20].

Hence, there could be an opportunity for designing mHealth apps that, to a larger degree, are designed from the user’s perspective, taking into consideration the everyday management of diabetes as well as contextual and motivational factors. Therefore, we engaged in a cocreation design process focusing on expressed user needs and context to address the challenges of long-term engagement. In the participatory design tradition, cocreation aims to develop solutions with end users acting as experts of their own experiences [21]. Users take on the role of designers by being given tools to create new ideas collaboratively with designers and other stakeholders [22], with researchers acting as facilitators [23]. Such a cocreation approach could reveal and address more fundamental issues faced by patients that might otherwise not be considered relevant [23] when only investigated from a clinical viewpoint.

### Objectives

In this paper, we present the results of this cocreation design process in the form of 6 themes and 2 prototype designs. The themes reflect common and recurring issues and solutions based on the users’ experiences with T2DM. One included topic is “actionable self-monitoring of blood glucose levels,” supporting users to reflect on their habits and make their own decisions. Another topic is “approaches to diabetes knowledge,” aimed at making diabetes more relevant and discoverable to the individual’s specific contextual needs. The prototype designs present 2 technological solutions for these 2 topics: an activity-based CGM app and a web-based personalized diabetes guide, including their user journeys and design rationales as well as examples of screen designs and interactions.

Overall, the results emphasize the need for positively framed content that encourages rather than uses the threat of impending complications [20]. The results indicate that structured SMBG is perceived positively and could empower patients with T2DM to make health-relevant decisions with positive long-term impacts on their own. The codeveloped solutions emphasize the need for flexible and individualized systems in terms of use, entry points, and intervention resources to address individual needs and preferences. These insights may be useful for designers working with T2DM solutions.

## Methods

### Study Design

**Figure 1:**
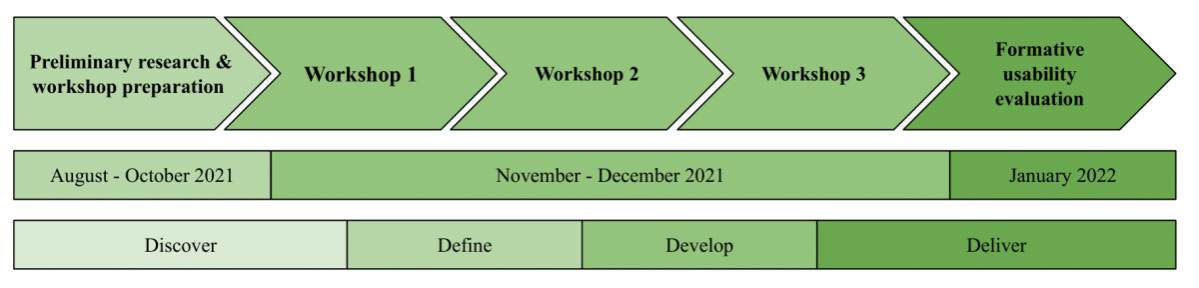
Overview of project activities and the timeline for activities.

A total of 3 workshops were organized in collaboration with the Danish Diabetes Association (DDA), with input drawn from the researchers’ prior work in diabetes technology combined with the long-term knowledge about patients with T2DM from the DDA. Subsequently, 2 prototypes were evolved and subjected to a series of formative usability evaluations for further maturation. This study has 5 distinct phases (Figure 1) based on the 4 phases of the diverging and converging double diamond model: problem discovery and definition, solution development, and delivery [24].

No restrictions were imposed regarding the possible solutions, and the scope and format of the solutions were left to the participants to determine through the workshops [15,16].

The workshops’ initial focus was on problem exploration of the diabetes space and opportunities. This was done by facilitating discussions centered around the participants’ personal experiences, journeys, and solutions with T2DM. Following this initial exploration, the problem space was assessed and refined by participants, and finally, 2 solutions were democratically chosen and explored based on the participants’ experiences, brainstorming, and discussions.

### Recruitment

For the workshops, we involved both patients with T2DM (*participants*) and a diverse group of *stakeholders*, including researchers, health care professionals, and designers. The recruitment material provided information about the purpose of the study and a link to the project’s website containing detailed study information. Each workshop started with a walkthrough of the workshop, its agenda, purpose, and aims and offered the possibility of asking questions. The recruitment criteria included persons with T2DM and those having experience with the SMBG, given that the patients with T2DM cannot know their blood glucose concentration unless they actively monitor it [8] and based on previous work showing that diabetes self-management education improves the quality of life by improving an individual’s decision-making [7].

Participants recruited for each workshop were encouraged to join subsequent workshops. Each workshop lasted 3 hours with built-in breaks. The requirements for stakeholders were work-related experience with patients with T2DM and knowledge of clinically relevant parameters. Stakeholders consisted of professionals working in the field of diabetes in the DDA and researchers from the Technical University of Denmark. Researchers from the Technical University of Denmark not facilitating the cocreation process participated as user experience designers.

### Ethical Considerations

Given the study’s technical and nonclinical aims, following guidance from the Danish National Center for Ethics and national law, ethics approval is not required for research involving surveys and interviews that do not include sampling biological material.

### Informed Consent and Participation

All participants provided informed consent to participate in the study by email or orally via phone before the workshops and were also reminded about the purpose of the workshop and their rights to withdraw. Collected data, such as notes and transcripts, were deidentified. Participants were compensated 200 DKK (approximately US $30) per workshop to cover transportation costs.

### Procedures and Facilitation

After setting up the space, with templates prepared and readied for distribution, the facilitator led the workshops assisted by a documenter taking notes. Facilitation was performed through a presentation with supporting slides, providing clear descriptions of exercises, timeframes, and goals. Participants were initially introduced to the workshops, overall project goals, specific goals for the day, and the agenda to ensure that all participants were on the same page. A warm-up exercise was carried out to introduce participants to one another and to create a comfortable and safe atmosphere, allowing participants to express their thoughts and opinions without fearing negative repercussions [25]. Workshop procedures followed the individual agenda for the day, with built-in breaks approximately every 45 minutes.

Inspired by Wróbel et al [26], the facilitator acted as a “third party” focusing on impartiality, equidistance, and fairness [26]. Even if a plan was prepared, facilitators were free to make changes along the way to address issues and to extend or shorten exercises as needed. Facilitators ensured that health care researchers and designers participated in all the groups, broadening the available perspectives.

### Preworkshop Exploration

**Figure 2:**
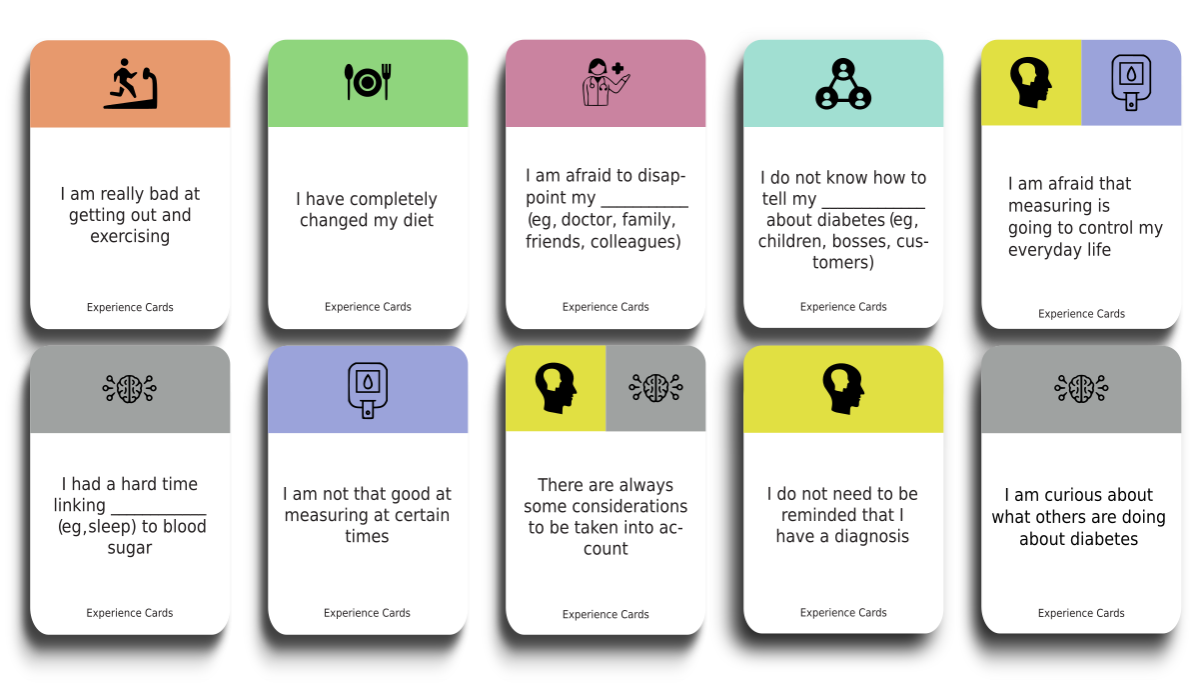
Examples of situational cards (translated from Danish to English).

On the basis of our prior works, a deck of cards was created containing 28 experience cards, referred to as situational cards, detailing different problematic situations that participants could find themselves in. The topics ranged from blood glucose, diabetes knowledge, personal issues, psychology, and diet to physical activity (Figure 2). Whenever possible, the cards were made generic so that everyone could provide their own context and meaning. Therefore, some cards contained blanks in which participants could add their own interpretations.

### Workshop 1

In the first exercise, participants discussed the situational cards in groups, creating an entry point for the workshop. The discussions initially aimed at sorting cards based on whether an issue felt relevant to the participants. Participants were encouraged to write on the existing cards or create new cards with their own issues or situations using the blanks provided. The second exercise consisted of storytelling, using previously created cards. In the third exercise, participants discussed and placed the cards on a horizontal timeline, with a vertical axis representing the importance of an issue at that time point. The timeline was purposefully made “vague” with no clearly defined starting point, ranging from before the diagnosis to the current day, with the only guiding mark being “time of diagnosis.” In the fourth exercise, the groups each presented their timelines with other groups freely commenting and providing feedback, promoting, and leveraging intergroup creativity [27]. The fifth exercise made the participants define overarching problems, issues, and pains using the developed timelines. The final exercise had participants brainstorming, discussing, and writing solutions to the issues previously defined by themselves, focusing on how they resolved or dealt with issues themselves or would deal with them now, with their present experience of living with diabetes for several years.

### Workshop 2

Before the workshop, we created a series of new cards based on the results from workshop 1 to be used as part of a design landscape game [28]. This “game” helped participants familiarize themselves with and add to the results of the previous workshop. The goal of the game was to discuss and group issues into thematic groups based on participant negotiations regarding the importance of the identified issues. Participants did this by placing problems on the board according to how important it is to them in general or personally, if conflicting views were present.

Participants then individually chose 1 problem from the most important area on the board. Each group then filled in a prepared template that explores who has the problem, what it is, and where or when it occurs. Afterward, the participants discussed possible solutions to the chosen topics in groups, using a provided solution template to ease the rough sketching of ideas and to ensure they became sufficiently concrete. Finally, the participants were asked to present their ideas in the plenum to collect oral and written feedback.

### Workshop 3

**Figure 3:**
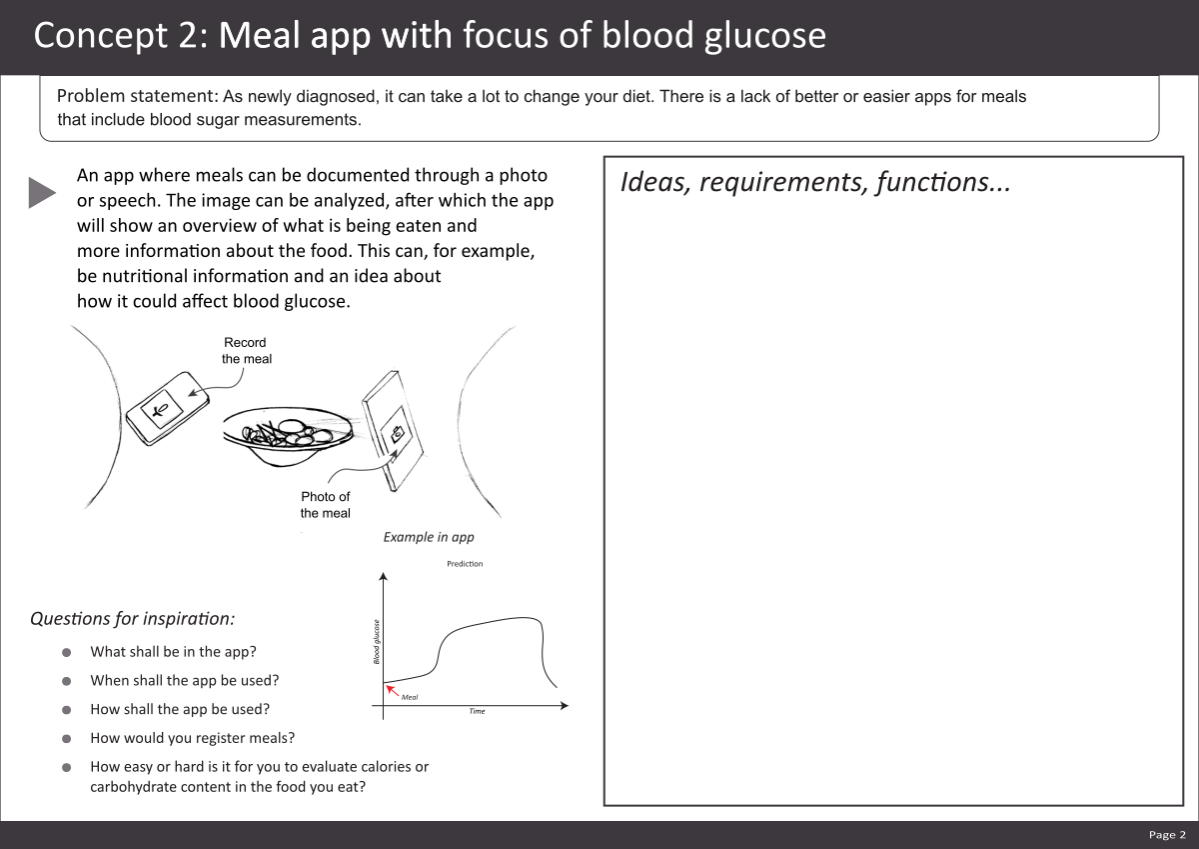
Example of a concept presented to the participants.

Before the workshop, we processed the previous outcomes to make some of the concepts more concrete. The workshop’s first exercise helped participants become familiar with these processed concepts facilitated as a “brainwriting” [29], generating ideas for each concept in groups. Each group was handed 9 pieces of paper, each with 1 possible concept for a chosen problem; an example is presented in Figure 3. Each participant started with one of the sheets of paper, individually reading and providing comments and ideas on the sheets. After a few minutes, participants exchanged sheets clockwise, also reading suggestions from the previous persons on the concept. When all the sheets were viewed by everyone, the participants discussed and voted on which concept they wanted to continue working on. This exercise was followed by a group discussion of the solutions with the most votes. The next exercise created a simple user journey to see what the journey (start, middle, and end) with the concept could look like.

This activity progressed to the final part of the workshop, where participants were split into 2 groups to prototype 2 different solutions. To allow researchers, designers, and participants to create and sketch their ideas, a wide variety of tools were brought to the workshop, including physical devices, digital solutions, options for more “social” aspects, and hybrid solutions.

### Formative Usability Evaluation

Following the third workshop, high-fidelity prototypes were developed by the authors for further validation and discussion in a series of usability evaluations with the workshop participants [30].

Each formative usability test was facilitated over the web and lasted 1 hour starting with an introduction by the investigators followed by testing of the prototypes. Participants with T2DM were given access to the high-fidelity interactive prototypes and were asked to “think aloud” [31] when actively trying out the prototypes [32,33]. At certain predetermined points, the investigators stopped the participants and asked them a set of semistructured questions on the design and user experience elements they had just been using. After 30 minutes, this process was repeated for the second prototype.

## Results

### Overview

A total of 20 participants with T2DM were recruited across all 3 workshops, with 7 participants returning for formative usability evaluation. In addition, 11 stakeholders participated: designers, researchers, and diabetes experts, including a nurse handling patients with diabetes.

Workshop 1 involved 13 participants (10 patients with T2DM and 3 stakeholders) and resulted in 10 stories; 3 timelines; and a collection of problems, opportunities, and wishes.

Workshop 2 involved 8 participants (4 patients with T2DM and 4 stakeholders) and resulted in the creation of 2 diabetes landscapes, prioritizing issues on both a general and personal level, and 9 concepts as follows: (1) an activity-based CGM with a companion app, (2) a meal support app with a focus on blood glucose and prediction, (3) a diabetes home test kit for earlier diagnosis, (4) an inspiration to everyday life with diabetes, (5) community-based motivation (physical activity), (6) individual motivation (physical activity), (7) an index of motivational and other groups, (8) knowledge center for diabetes, and (9) an introduction to diabetes with structured blood glucose measurements. Multimedia Appendix 1 provides detailed descriptions.

Workshop 3 involved 10 participants (6 patients with T2DM and 4 stakeholders) and resulted in 2 concepts to develop further: (1) an app for blood glucose monitoring using CGM and (2) an introduction to diabetes.

Finally, 7 participants were included in the formative usability evaluations, resulting in several improvements relating to content, ordering, and usability issues.

Following the workshops and formative usability evaluations, a thematic analysis was conducted based on the created artifacts, field notes, and formative evaluation transcripts. We identified the following themes: (1) diabetes care, (2) diabetes knowledge, (3) glucose monitoring, (4) diet, (5) physical activity, and (6) social aspects. In subsequent sections, we explore these thematic insights, starting with an overview of the timelines created by participants, followed by an in-depth exploration of the developed concepts, their user experience, and rationale.

### Timelines

**Figure 4:**
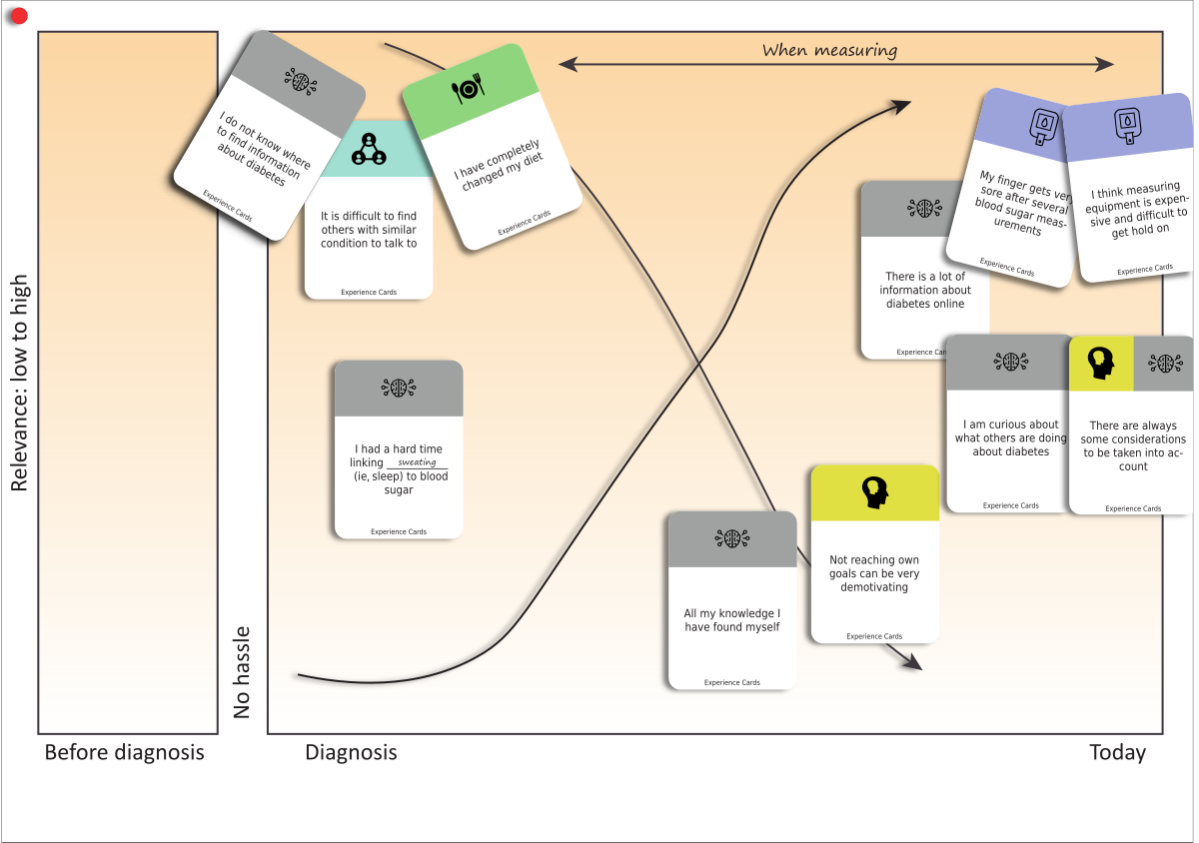
A timeline created by participants. Hand-drawn lines indicate different perspectives over time when measuring blood glucose.

The timelines created by the participants were diverse with respect to the subjects covered; some participants put emphasis on motivational issues at the onset of diagnosis and others later, reflecting their individual experiences. Most participants agreed that finding information was a big challenge at the time of diagnosis, with some stating that dietary changes or motivation to exercise were also an initial concurrent issue. An example of a timeline is presented in Figure 4.

### Themes

#### Diabetes Care

Participants voiced several concerns about how diabetes care is handled in Denmark, ranging from a perceived lack of specific diabetes knowledge among general practitioners (GPs) to inadequate time available for consultation. Their concerns in this area extend beyond the GPs to the local municipalities that have different measures, strategies, and perceived levels of care to aid patients with T2DM or may lack adequate initiatives or patient education, leading to a perceived treatment gap. This was, for example, reflected in the discussions among participants pertaining to the ease of attaining strips for blood glucose measurement from the health care system through different municipalities. The educational material provided was brought up, because for some participants it was irrelevant, lacking actionable elements, outdated, or not working.

Participants generally critiqued physicians and municipalities that were restricting access to blood glucose measurement devices, which many participants thought should be available to all patients with T2DM (policy requires the municipality to approve requisitions for diabetes devices made by the GP). Participants cited this as especially problematic, given that such measurements can provide key insights into diabetes and how it affects them. One participant even stated, “I think everyone should have a device for continuous measurements the first 14 days [after diagnosis], and focus on insights and connections,” with “connections” referring to how a person affects their blood glucose concentration through actions taken and how it affects them in return. Several participants highlighted stories about how blood glucose insights helped them: “I start feeling ill if my blood glucose is too high or low. Too high and I become hot, too low and I start getting hungry” and “measuring blood glucose does not make one feel more ill, quite the opposite, because you can then do something about it.”

Regarding perceived knowledge among GPs, some noted, “I always need to educate my physician” and “I don’t think the physician has a sense of diet, but the nurse often does.” However, others highlighted positive experiences: “When I visit my physician, I always get a good talk.” Participants emphasized that a good relationship with GPs is important for patients with T2DM, whereas a lack of a good relationship can be detrimental.

#### Diabetes Knowledge

The participants highlighted that there are many approaches to finding information and that it is not uniformly available for all. The approaches included the following: GPs, nurses, the DDA, “Doctor Google,” Facebook diabetes groups, blogs, more experienced mentors, and books or flyers. Issues ranged from too little to too much information. Examples may be receiving too much information and having “to sort wheat from the chaff” or not receiving enough information, “I lacked an introduction to diabetes.”

This often leaves the burden of information search on the patients with T2DM themselves and extends to the type of information found as well, especially over the web. Information may be incomplete, lack context, or emphasize negative aspects and may cause hopelessness and frustration in the worst case, presenting a potential barrier to further self-management [20,34]. A specific example may be the possible complications of diabetes, making patients with T2DM feel overwhelmed and discouraged owing to a sense of hopelessness not being able to avoid comorbidities. Even when knowledge is found, its format or content may make it difficult to translate it into specific behaviors or actions.

Another important point of discussion was about balancing life and diabetes, as stated by 1 participant: “Life isn’t just about diabetes.” An acceptable equilibrium must be found between diabetes management and living life. For some, this meant finding ways of exercising that provide joy, such as enjoying nature, or accepting having “off days” with less focus on diabetes. Finding this balance was perceived as a crucial aspect for the participants’ quality of life in the long term.

#### Glucose Monitoring

Measuring blood glucose was cited by all participants as a positive experience, resulting in insights into how their body works. Some explained that they previously participated in a structured program of blood glucose measurements, where before a measurement, a guess was made about the blood glucose, and that values differing from the expected promoted reflection on the cause, leading to insights and new knowledge. The participants also discussed that more knowledge could be useful, especially related to blood glucose targets. One participant stated, “How dangerous is it that my blood sugar is too high and how long can the body handle it?” in the context of daily spikes in blood glucose and what impact it may have on complications in the long term.

Some participants had imported their own CGM from outside Denmark and used it for a period to generate individual insights into diabetes. Although participants were generally happy with structured SMBG, they nevertheless preferred CGM solutions for not having to bring SMBG devices, the discomfort related to SMBG, and a limited number of measurements per day, which can be costly and difficult to acquire in Denmark. Access and cost of use were generally a key concern for both structured SMBG and CGM.

After using structured measurements for a period, many transitioned to more sporadic measurements on an as-needed basis or to check the effects of exercise, certain meals, or hunches. Symptoms such as tiredness, feeling unusual warmth, or blurred vision were among the symptoms described and coupled with high blood glucose levels. However, others did not find such correlations, as they were unable to notice symptoms of high blood glucose levels.

#### Diet

Participants had very different approaches to diet; some had changed their diet radically, and others less so. Participants talked about creating their own diet and exercise habits. Changing diet requires significant effort at the onset of diagnosis and gradually lessens over time as new habits are formed. Initially, knowledge about diet was sought from multiple sources, some receiving information from physicians and nurses, others through diabetes cookbooks, Facebook groups, or merely through trial and error through blood glucose measurements.

Some participants created “rules” for when to eat and how to balance diet with exercise. These specific rules were based on subjective experiences and insights into their own body. Blood glucose measurements were mentioned as a great tool to assess certain foods and to gain insights that can be used for subsequent decision-making.

#### Physical Activity

The main issue with physical activity is motivation but extends to difficulty correlating exercise with long-term blood glucose level improvements and a lack of knowledge on the impact of specific factors of exercise; that is, the importance of intensity or heart rate.

Several participants reported that they had created their own solutions to motivate physical activity, ranging from gym membership to team sports, and finding alternative ways to enjoy daily exercise. Some maintain motivation by committing to others, such as a team sport, a commitment to a group of peers or even to oneself. More personal motivations include exercising to see new places or enjoying nature. Both physical groups and web-based groups were mentioned as providing motivation to engage in exercise, with competitions as an additional motivation for some. Perceptions of motivational factors generally vary, with the consensus that no single motivational solution will suit everyone.

#### Social Aspects

Several social aspects of diabetes were mentioned, especially in relation to motivation, experience sharing, and knowledge. Local groups and Facebook groups are considered good sources of knowledge, inspiration, and support. The collective knowledge from such groups, especially larger web-based communities, is perceived as useful, where posed questions often generate helpful answers whether the question relates to newly diagnosed or more specialized problems.

In-person physical meetups for exercising or cooking were liked by some participants but were also perceived to have challenges. Given the individual nature of diabetes and the broad segment of affected people, geography, social status, and the wide variety of goals and commitment between people, it can be difficult to find like-minded peers. For such groups to work, emphasis was placed on aligning goals and being comfortable with those involved. Making commitments to groups, another person, or even oneself was perceived as a positive way to enhance motivation. The individual needs of patients with T2DM were reflected in differences in opinions about the level of commitment and content in such groups. Some were inspired by others and benefited from external commitments, whereas others found motivation through competitions or the joy of simply carrying out activities socially.

### Designing mHealth Technology for Diabetes Care

On the basis of participants’ majority votes, work continued with 2 prototypes: one focused on the themes of blood glucose monitoring, diet, physical activity, and social context and the second focused on the themes of diabetes care and diabetes knowledge, while also incorporating elements of glucose monitoring, diet, and physical activity.

#### Activity-Based CGM (Prototype 1)

**Figure 5:**
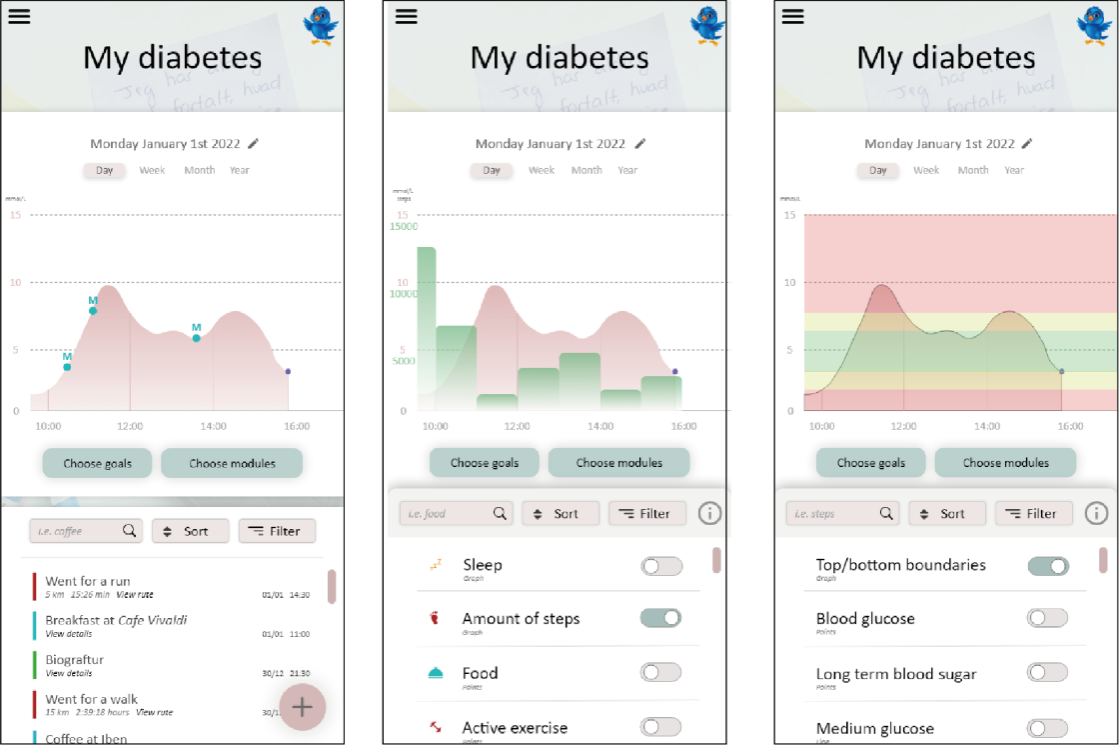
The app’s home screen and the “My diabetes” overview with several meal menus (left), recorded steps (middle), and top and bottom boundaries (right).

In prototype 1, participants created a metaphorical timeline [18] representing a person’s daily life with diabetes (ie, “my diabetes”) through the use of CGM data and events. It initially shows only CGM data, but users can continuously modify and populate the timeline with different tracked events through “modules” representing activities related to diabetes. The most important activities identified by the participants were diet, physical activity, sleep, and context (eg, location and activity recognition). Choosing a module prompts the system or the user to collect data related to that module and populate the aforementioned timeline with individual or aggregated events. The events and the timeline are clickable and allow the user to extract additional information; for example, event details and contextual information, such as where and when an event occurred. An example of such a timeline and events is presented in Figure 5. Users can modify the timeline using event filters, different time periods, and graph overlays, reflecting individual goals such as long-term blood glucose management. On the basis of data collection, the system may provide suggestions and feedback on the data or highlight certain data and patterns. The central idea is to empower the patients with T2DM to identify trends and increase awareness of factors affecting blood glucose level, which can be used as a basis for reflections and for making decisions [35].

**Figure 6:**
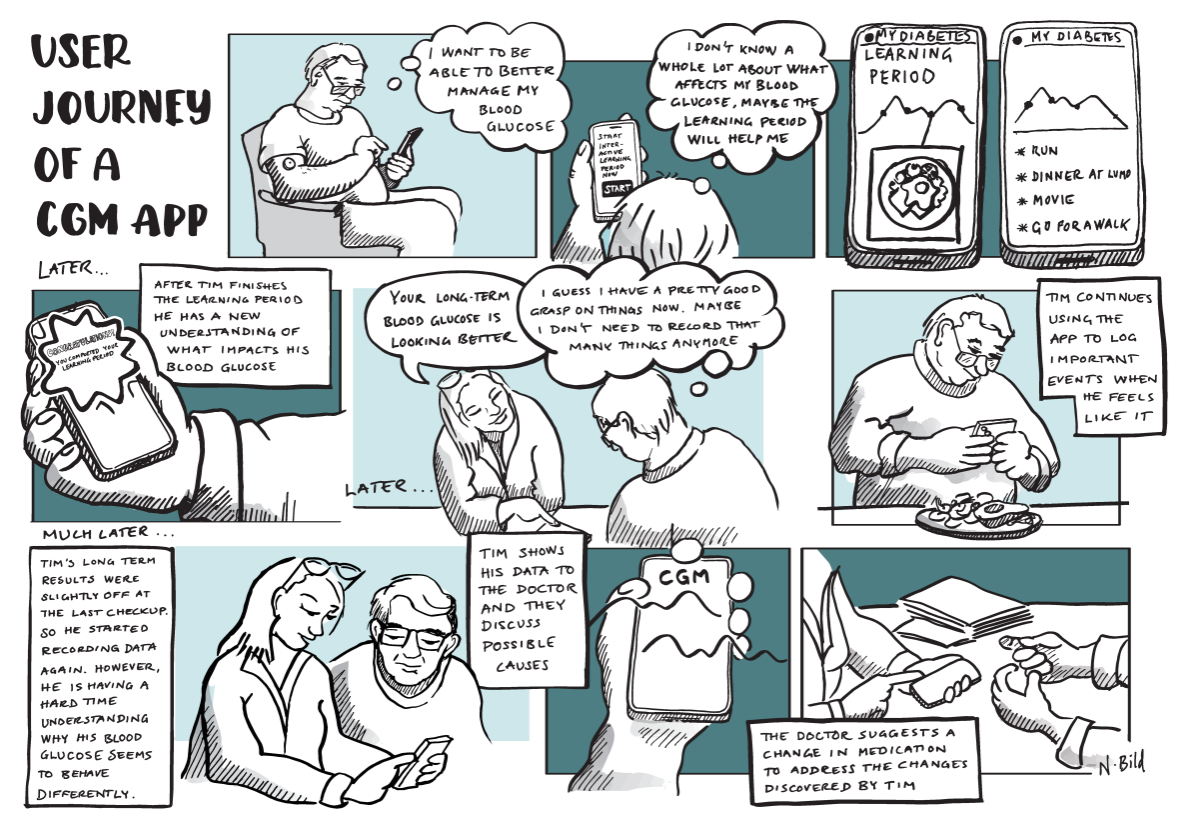
User experience storyboard for a continuous glucose monitoring app.

To keep the app relevant over time, several distinct phases of use were identified by the participants, which can be summarized as follows: (1) the initial use and learning period (with CGM), (2) the active measurement period (with CGM), and (3) the support tool period (without CGM). These are reflected in the user experience storyboard of Figure 6, which shows the initial learning period, the transition support tool period, and a return to active measurement.

The app will initially provide an easy entry point for self-exploration, allowing the patients with T2DM to start at their own pace and later add more activity types with the benefit that they can focus on learning about the effect of individual factors; for example, the effect of diet on blood glucose, exercise, and sleep. Breaking the learning process into distinct phases of focus reduces the risk of fatigue from burdensome data collection because users only collect (manual) data for the active modules based on their current focus. In addition, automatic data collection aims to reduce the data collection burden and to provide contextual information through mobile sensing.

The active measurement period allows the user full access to different activity modules and a choice of how to use them actively to facilitate self-management, providing supporting suggestions. Participants pointed out that over time (a span of years) and as the disease progresses, it might be necessary to have several shorter active periods; for example, to address changes in their condition, new goals, or recommendations by GPs.

In the support tool period, participants envisioned less frequent use of the app, with data occasionally being logged in by the users, enhanced with continued automated collection of, for example, physical activity and sleep data. Users can use the app to review old data and notes and continue adding observations from, for example, SMBG or HbA_1c_ measurements from the GP.

#### Diabetes Guide (Prototype 2)

Across their individual journeys with diabetes, many participants felt they could have used a better introduction to diabetes, especially after having experienced structured SMBG (ie, measurements with a certain timing and frequency) to facilitate learning. Therefore, a web-based guide to diabetes was developed as a prototype centered around blood glucose, providing multiple entry points and opportunities for an individualized approach to self-management.

Participants imagined this guide to use structured SMBG with supporting educational material. The content of the guide is based on high-quality videos, with more information available in text format and links to additional relevant verified sources. A patient with T2DM can then choose the level of detail required, allowing both a low-barrier entry point (eg, videos) and more in-depth information (eg, text and links to other educational resources), when desired. The navigation of the guide was imagined to adapt to an individual’s perceived situation with diabetes, always providing content relevant to the individual. The main categories to organize content around are (1) the nature of diabetes, (2) blood glucose level, (3) physical activity, (4) diet, (5) motivation, and (6) inspiration.

**Figure 7:**
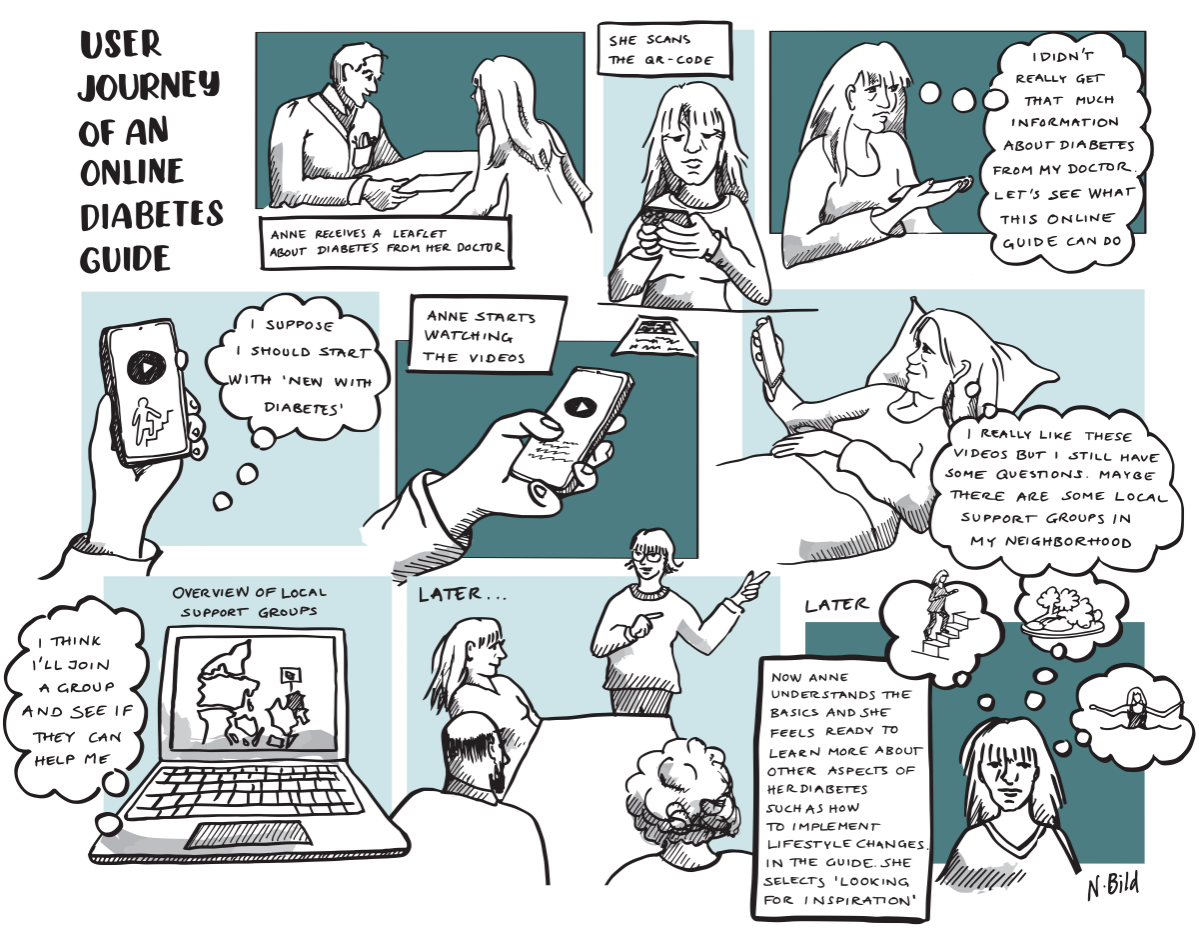
User experience storyboard for a web-based diabetes guide.

The overall journey through the solution starts with the GP upon diagnosis, where an easy-to-read, brief folder is provided, encouraging the use of the diabetes guide accessed via a QR code. When entering the website, a video will start explaining the structure of the guide, and the patient with T2DM can then identify with one of the several “situations” and be forwarded to the relevant content. In the workshops, these situations were initially imagined as 3 concrete scenarios ranging from newly diagnosed patients with T2DM to those experienced with diabetes but subsequent formative usability tests suggested additional scenarios. For the selected scenario, a video is also played, providing more specific situational information. Newly diagnosed users could be advised to start measuring blood glucose, although the individual can always choose the order in which educational material is used. As the patients with T2DM gain more experience, their perceived situation may change and they can choose a new scenario to see content that is more relevant. This journey between the perceived situations is presented in Figure 7.

**Figure 8:**
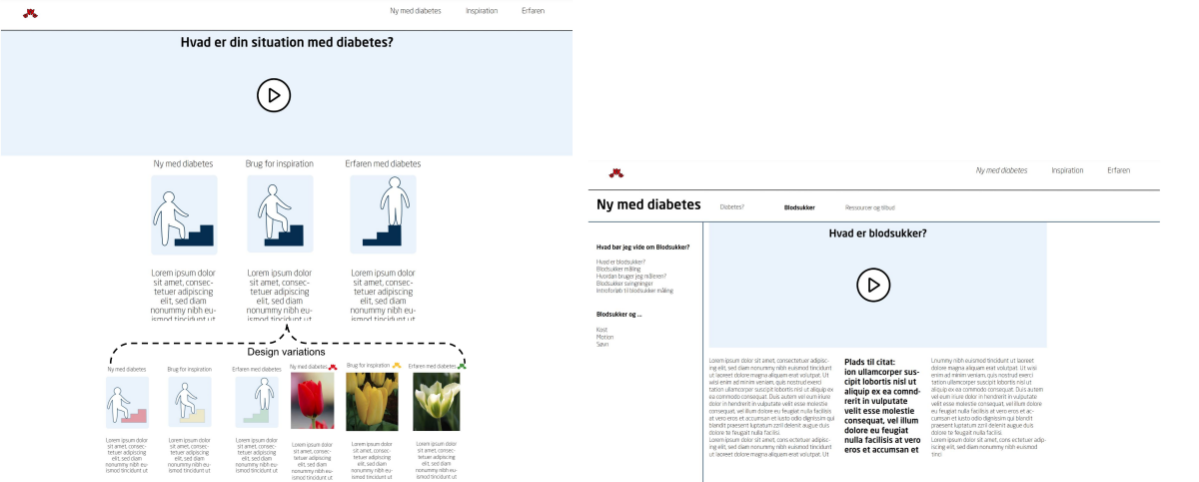
Prototype 2 (in Danish): the left screen shows the guide’s landing page with different entry points described through perceived situations and presented to the users by a video explaining the situations and the guide’s structure, including designs visualizing the perceived situations. The right screen shows an example of what the guide’s content might look like with links to other content and useful pages, such as an overview of local resources and offers.

Although participants thought that this solution would have been especially relevant for them when they were newly diagnosed, they also liked the idea of being able to go back and find new videos and material relevant to their current situation. This led to discussions about possible ways of individualizing content beyond the guide’s initial scenarios, where more personalized content could be suggested to users having created an account on the site, based on previous consumption of content, needs, and personal preferences. Accounts would further enable the patients with T2DM to keep track of their use of the guide through bookmarks and history. Participants further emphasized that providing curated links to other resources, communities, and solutions as well as providing an overview of available options outside the initial guide would be useful. This would act as a hub for curated diabetes information and resources that the patient with T2DM may choose to engage with based on their preferences. An overview of the website structure is presented in Figure 8.

## Discussion

### Principal Findings

It has previously been found that the needs of the patient might not align with what is considered clinically relevant [16], as also revealed in this study. For example, some participants argued that they would prefer not to include monitoring of body weight because this would incite a “culture of weight loss.” Although the topic of weight loss was briefly discussed in the first 2 workshops, it was generally not a topic that the participants focused on. Even though modest weight loss has shown significant benefits in diabetes [36-38], participants argued that focusing on weight loss would take the attention away from what they saw as the key issue, namely monitoring blood glucose levels.

Although the clinical value of measuring blood glucose levels in patients with T2DM is a highly debated topic with diverging points of view [39-42], this study found that patients with T2DM perceive these measurements and the insights they provide as valuable. Throughout the workshops, a variety of devices and measurement types were discussed. The participants reported that measuring blood glucose levels in a structured and frequent manner using SMBG was useful for associating behaviors with changes in blood glucose levels. This supports earlier findings that patients with T2DM perceive structured SMBG as useful [40,43], especially with more frequent measurements. Tomah et al [40] reported that a higher SMBG frequency was associated with larger improvements in weight and HbA_1c_, suggesting that the frequency of measurements may have a direct effect on clinically relevant factors, with 4 to 8 measurements per day yielding the best results [40]. Similarly, Mannucci et al [43] found that the effects of SMBG measurements are better with structured SMBG programs and when the GP uses the collected measurements [43].

Although participants found SMBG useful, they noted limitations to this approach, such as having to carry a device, cumbersome to use in certain contexts, and the lack of insight between measurements. Therefore, participants argued that they would prefer CGM over SMBG, with some drawing inspiration from the technology used by patients with type 1 diabetes mellitus. Even though few participants had experience with CGM technology, they perceived CGM to ease data collection and provide the highest fidelity of data for self-management. This perception is supported by clinical evidence showing that the use of CGM has shown small but clinically relevant reductions in HbA_1c_ levels [44], along with an increased awareness of the impact of diet, exercise, and medication on glycemic control and weight control [41]. This integration of different blood glucose factors is also evident in the design of the prototypes showing how the insights from CGM may be enhanced by the integration of diet, exercise, education, and counseling.

### Prototypes

Both developed prototypes include support for education in the form of increased awareness gained from blood glucose measurements. Although they could be seen as mutually competing, as one focuses on introductory education and the other focuses on finding patterns [35], participants indicated that the prototypes complemented each other by addressing different needs. Experiences explored in our workshops indicate that these insights can be used by the patients with T2DM to implement personalized lifestyle changes through increased self-awareness [45] and informed decision-making. Enabling patients with T2DM to make clinically relevant decisions could potentially reduce the reliance on GPs for basic insights, enabling GPs to focus on other needs of patients with T2DM.

Compared with other mHealth apps, the design of prototype 1 is notable for several reasons. First, it provides a unique perspective on how users want to use mHealth over time for T2DM through the different use phases identified. Second, its design implies that if a user is not using the app, this means that they might have reached a goal. This suggests that attrition as a measure of failure in a traditional sense may not necessarily make sense for this type of mHealth app. Third, it emphasizes the user as an active part of the intervention rather than simply as an intervention recipient. Many authors note that long-term engagement remains a key challenge in mHealth [14,46] and is reflected in the high attrition rates seen in many mHealth apps [13,14]. However, the rationale for prototype 1 suggests that such periods of low or no use can actually be a preferred outcome of using an mHealth app. Indeed, the aim of both the learning and active periods is to enable patients with T2DM to draw insights that help them improve glycemic control to a point where the use of the app is not necessary, thereby transitioning from an active (high use) period to a support period (low or no use). Given the chronic nature of T2DM, support (low use) periods may last for a substantial period, perhaps until the patient with T2DM decides or is prompted to start another high-use period. Such an active period may be prompted by the patient’s GP in response to passive data collection or by a self-initiated need for change [34].

Discussions among the participants and the timelines created (Figure 4) emphasized that the usefulness of the components changed over time. For example, although structured SMBG or CGM may be useful for some time, it may not be necessary all the time. We speculate that this may be because of the perception that various app or intervention components are not valuable enough when the blood glucose level is within the individual target range to offset their resource requirements [47]; that is, data collection, monitoring, costs, and other discomforts from use. These findings suggest that although mHealth technology could serve a permanent role in the lives of patients with T2DM, many may prefer to use such solutions intermittently or for finite periods unless serving specific needs. These observations are in line with previous findings from Klasnja et al [34]:

The design of tools for diabetes that support long-term engagement should allow periods where the individuals can suspend use of one or more features of the application.

This consequently calls for more exploration of the users’ needs and the user experience delivered by digital solutions over time [48].

In the design of prototype 1, participants argued that they preferred to be an active part of the intervention rather than simply a recipient of the intervention components. This was reflected both in the group discussions and the different approaches to diabetes self-management designed by the participants. Rather than being presented with a limited set of options, participants preferred to be presented with data and long-term trends (in some cases, summaries) that they could act upon. The final design and intended use of prototype 1 resembles the Stage-Based Model of Personal Informatics Systems [35], in which a reflection component leads to behavioral change. Furthermore, we found that the participants wanted the ability to tailor the system to their personal needs with high flexibility. For example, a newly diagnosed patient with T2DM would need time and support to reflect on their own data, to experiment, to identify trends, and to learn how to act upon these in a structured way when first using the app. This process of learning through SMBG feedback, that is, measurements and reflection, has previously been seen as a core component of how a well-controlled patient with T2DM learned to successfully manage T2DM [49].

While also emphasizing the value of structured SMBG through educational content, prototype 2 is focused on facilitating self-exploration that aims to help users find approaches to diabetes self-management that work for them and in their own context. One notable aspect of the design of the guide was that multiple approaches to self-management behaviors should be included, for example, in relation to diet, physical activity, and blood glucose measurements. Following the review by Skinner et al [20], we see significant potential in a guide containing a catalog [50] of “interventions” that users can choose from and put together in ways that serve their own needs and preferences. Participants noted skepticism toward the idea that 1 “solution” could serve every need or preference, a notion also supported in mHealth literature [34,51]. Participants also noted that not everyone would necessarily be interested in using purely digital solutions (such as prototype 1), making it useful if the guide also contained nondigital interventions, such as workshops and classes.

**Figure 9:**
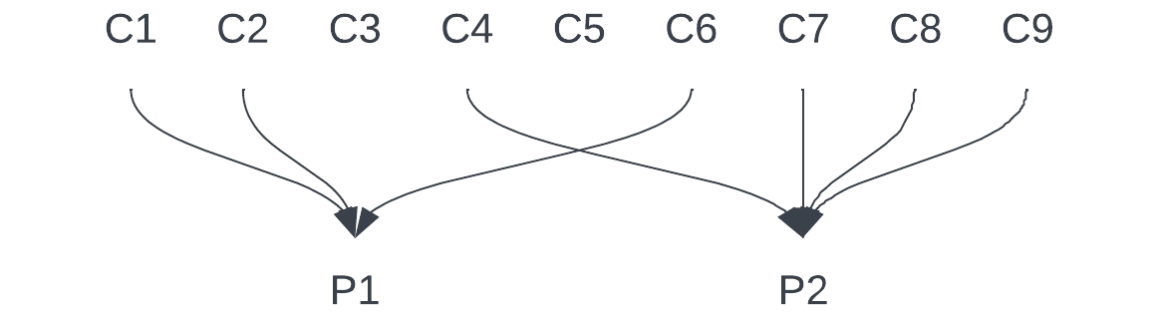
A simple overview of how the developed concepts (C) have contributed to the final 2 prototypes (P).

In addition to the 2 prototypes selected and designed by the study participants with the aid of the researchers, several other concepts were discussed although not chosen for further development (Multimedia Appendices 1 and 2). However, it is worth noting that many elements from these other concepts were incorporated into the 2 final prototypes: prototype 1 combines ideas from concepts 1, 2, and 6 and prototype 2 combines ideas from concepts 4, 7, 8, and 9. However, both are still centered around blood glucose measurements and the insights they provide (Figure 9; a detailed overview can be found in Multimedia Appendix 2).

### Additional Insights

During the study, additional discussions emerged on approaches to self-management based on participants’ individual habitual implementations of activities such as diet and physical activity. *The participants generally had very individual journeys with diabetes*, depending on the circumstances and personal preferences for self-management behaviors. This was reflected in their level of motivation at different stages and by what motivates the individual to engage in self-management behaviors. Concerns were raised several times whenever generic “one-size-fits-all” ideas came up and are also reflected in the journeys, motivation, implementation of habits, and our final prototypes focusing heavily on individualized use.

Discussions among participants showed a multitude of motivations ranging from fear of comorbidities, wanting to avoid medications, experiencing a positive or negative change, competition, and intrinsic enjoyment of activities, to individual and group motivation. Although each of these may be motivational factors for some, they might pose a barrier to others. Rise et al [52] describe 4 motivational factors to maintain changes: support from others, experiencing an effect, the fear of complications, and the formation of new habits. Although these factors are also reflected in our results, we add that the combination of individually perceived motivational factors seems to vary greatly among people. Contrary to the study by Rise et al [52] that concludes that knowledge leads to no changes if diabetes does not appear scary, our study indicates that although this may motivate some, it may present a discouraging barrier for others. Wishes and comments made by participants suggest *instilling fear is not beneficial or useful in promoting motivation for self-care.* Recent research further supports this notion, indicating that it could endanger mental health [53], with anxiety being a factor related to diabetes fatigue [54].

Furthermore, *poor communication with GPs, as also reflected in many comments from the participants, has likewise been linked to diabetes distress* [20]. According to Skinner et al [20], some GPs may attempt to “use the threat of impending complications or the need to use insulin in those with type 2 diabetes as a means to try and motivate self-care.” Although not a direct finding of this study, comments made by the participants indicate that some web-based literature and educational materials also use this method of instilling fear. Our results also support the findings of Skinner et al [20] regarding the potential downsides of limiting opportunities to explore personal preferences and how to best manage diabetes in the long term.

Despite participants’ differing opinions on which approaches are preferable for lifestyle changes, *the participants universally found structured SMBG useful for gaining insights into how actions affect blood glucose level and learning how to affect one’s blood glucose levels*. We noted that participants’ experiences generally align with those found by Pludwinski et al [45] with regard to self-activation and SMBG, increasing the self-awareness of behaviors and diet. Participants highlighted the importance of performing blood glucose measurements in a structured way as it provides them with key insights and lessons about diabetes they have not been able to obtain elsewhere. One participant stated that insights allowed defining their own rules for self-management: “I have my own blood glucose rules (my max is 8).” Another stated, “It [SMBG] ensures patients with T2DM have to know how different food items affect their blood glucose, as the effect is different from person to person.” Several participants suggested that structured SMBG should be embedded in the general introductions to diabetes, where newly diagnosed patients learn what affects their blood glucose levels and how they can impact it. This provides encouragement and self-empowerment, achieving insights unlocked by a period of structured SMBG. The value of using SMBG specifically with newly diagnosed patients with T2DM is further supported by a recent cohort study noting that SMBG improved glycemic control in newly diagnosed patients with T2DM [55].

Our results show that patients with T2DM are interested in highly personalized systems, providing them with choices that match their specific needs and wants in the current situation. Our findings indicate that *supporting and empowering patients with T2DM to discover and implement their preferred approach to self-management may reduce barriers to long-term management* and improve quality of life [34]. Leveraging differences in motivation, approach, and preferences through individualization of digital solutions, rather than using a generic approach, seems key.

Structured SMBG and CGM seem to play a key role in this process, supporting patients with T2DM in making lifestyle decisions for themselves when health care professionals are not available. *Patient-provided SMBG data through our first prototype could also potentially support the clinical care process with health care professionals* [56] to the extent that the patients with T2DM would be willing to share data with, for example, their GP without losing agency.

Regarding the generalizability of our findings, we noted a significant number of similarities between our results in Denmark and those reported by Klasnja et al [34] in the United States. For example, Klasnja et al [34] identified 4 areas in which technology could play a supportive role in diabetes: (1) understanding the new disease, (2) responding to changes in times of stability, (3) improving communication, and (4) tailoring to individual motivations and needs. Not only are these roles mostly fulfilled by the codeveloped prototypes, but also the technology described, features included, rationale, and suggested user experience over time are quite similar and almost identical at times, despite a difference in the methods used to arrive at these findings. For example, Klasnja et al [34] described tools facilitating tracking of multiple glucose-affecting factors to support learning about interactions between these factors using visualizations and pattern recognition reminiscent of the design of prototype 1. Other similarities (to mention a few) include the development of user needs over time, the role of SMBG, use patterns, and the perceptions of motivational factors [34].

### Limitations

Despite following the national COVID-19 pandemic guidelines, we noted that there was a high dropout rate among signed-up participants across the workshops, which may have been because of an increase in infections during this particular period. The limited number of only Danish workshop participants and a lack of wider cultural and global demographic diversity may reduce the generalizability of the findings. Participants in general seemed highly autonomous in their handling of diabetes and may not represent the wider population of all the patients with T2DM. This may also limit the generalizability of the developed prototypes.

Several methodological reflections can be made regarding facilitation. We chose to use soft facilitation focusing on the content rather than following rigid timetables for the workshops. This resulted in some methodological deviations to ensure that the workshops were completed on time, allowing certain discussions and exercises to run longer while shortening other exercises to accommodate.

The recruitment criteria may have introduced a bias pertaining to the measurement of blood glucose level, as experience with SMBG is a requirement for recruitment. Even if based on the participants’ own experience, this could have limited the scope and biased the voting of the developed solutions, as significant focus revolved around the measurement of blood glucose level rather than on control of blood pressure and lipid level. Consequently, we note that further work is necessary to ensure that the prototypes are functional for those not experienced in monitoring blood glucose levels.

The GPs are not explicitly included in this study because of concerns about introducing an “authority” in our workshops. Given the criticism and discussion of both policies and the role of GPs and municipalities, the inclusion of GPs could have caused an uncomfortable atmosphere; however, it may have resulted in less innovation potential and a lack of reflection from a clinical perspective.

Finally, because we did not aim to test functional prototypes, a follow-up study is necessary to verify the long-term effectiveness and to assess the perceived usefulness of the solutions.

### Conclusions

This study presents the results of a cocreation process involving a group of patients with T2DM and relevant stakeholders. A total of 3 workshops and 7 formative usability evaluations were conducted involving 20 patients with T2DM and 11 stakeholders. The cocreation process resulted in a detailed understanding of 6 diabetes-related themes: diabetes care, diabetes knowledge, glucose monitoring, diet, physical activity, and social aspects. Our results support the use of structured SMBG and CGM as useful and desirable tools and emphasize the desire of patients with T2DM to be empowered and active participants in their own treatment.

Two differently scoped prototypes were conceived: (1) a CGM app to support self-learning and (2) a web-based, personalized media-rich diabetes guide. The needs of patients with T2DM are often personal and change over time, shaped by individual experiences. This is reflected in the prototypes by focusing on user agency and with distinct use phases that adapt to the current context.

Our results may be especially useful for designers of mHealth technologies because they provide insights into the different and changing needs of patients with T2DM.

## Appendix

Multimedia Appendix 1 Overview of brainstormed concepts from workshop 2.

Multimedia Appendix 2 Overview of how the developed concepts contributed to the final 2 prototypes.

## Abbreviations

CGM: continuous glucose monitoring
DDA: Danish Diabetes Association
GP: general practitioner
HbA_1c_: hemoglobin A_1c_
mHealth: mobile health
SMBG: self-monitoring of blood glucose
T2DM: type 2 diabetes mellitus

## Data Availability

The data sets generated and analyzed during this study are not publicly available because the participants did not give their consent to share nonaggregated data.
