# Supplementary material for: Exploring Patient Needs and Designing Concepts for Digitally Supported Health Solutions in Managing Type 2 Diabetes: Cocreation Study
Source: JMIR Form Res. 2023 Aug 25;7:e49738. doi: 10.2196/49738 (PMC10492168; doi:10.2196/49738)
Supplement: Multimedia Appendix 1 [file formative_v7i1e49738_app1.docx]

**Multimedia Appendix 1**

Workshop 2 resulted in several concepts being developed as potential solutions to the problems the participants prioritized. These were brainstormed as direct solutions to many of the problems mentioned in the above themes and resulted in the following concepts being Imagined:

1. Activity based CGM with companion app
2. Meal support app with a focus on blood glucose and prediction
3. Diabetes home test kit for earlier diagnosis
4. Inspiration to everyday life with diabetes
5. Community based motivation (physical activity)
6. Individual motivation (physical activity)
7. Index of Motivational and other groups
8. Knowledge center for diabetes
9. Introduction to diabetes with structured blood glucose measurements

1) Concept 1 is envisioned to be an app combining CGM with contextual information about diabetes related behaviors. The basic idea is to enable broad data collection and easy analysis of trends with CGM data to create insights and self-reflection. Emphasis was put on the CGM device being affordable with electronics and needles being separated.

2) Concept 2 is envisioned to be a meal support tool, where meals are documented by photos and can be analyzed to displays nutritional information for the meal. Additional features include decision support elements in the form of predicting the blood-glucose based on the image, such that the app provides a best-guess on how the full meal will effect blood glucose allowing the PwD to adjust planned food intake if needed.

3) Concept 3 aims to address issues of late and sometimes coincidental discovery of diabetes and was inspired by other Danish healthcare screening programs. Here, the idea is a home test kit for T2DM, with instructional and motivational material addressing why taking the test is a good idea. The core concept is thus a low-effort, low barrier approach to early discovery of pre-diabetes and undiscovered T2DM.

4) Concept 4 relates to searching for, sharing and finding trustworthy inspiration to everyday life with diabetes. Core to the solution is the idea of forum with easily search-able content, to enable broad sharing of knowledge and approaches to everyday life with diabetes. Here PwD can share problems, victories, inspiration, local offers or promote local initiatives and easily locate information relevant to them.

5) Concept 5 is based around motivation and staying motivated in groups by making commitments to the group/community. Based on participant experiences this is envisioned as a hybrid physical/digital solution, supporting both groups based on common activities and when they choose to meet virtually.

6) Concept 6 is conceptually about finding reasons to engage in physical activity and inspiring people to engage in more physical activity, with the idea to provide inspiration, activities and travel destinations where one can increase physical activity while seeing new places. Statistics, gamification elements, and competitions were among the suggested features.

7) Concept 7 is suggested as a possible solution to reduce barriers to joining diabetes groups or finding a mentor and is envisioned as an index of local groups/persons. The solution aims to ease the process of finding groups with similar lifestyles, needs, or wants based on geographical and other requirements, reducing the effort needed to find like-minded peers.

8) Concept 8 is a platform where knowledge and diabetes resources can be gathered with a focus on validated information. Inspiration was drawn from a Danish health website "sundhed.dk", but with a focus on aspects of diabetes and the questions that naturally arise from diagnosis and forwards. The platform is curated; knowledge is importantly to be verified by experts and credible sources made available to users to increase transparency and trustworthiness.

9) Concept 9 is an introduction to diabetes, centered around structured blood glucose measurements, where participants receive an adequate introduction to diabetes with blood glucose measurements to support insights into diabetes and how it impacts the individual.
