# Supplementary material for: Exploring Patient Needs and Designing Concepts for Digitally Supported Health Solutions in Managing Type 2 Diabetes: Cocreation Study
Source: JMIR Form Res. 2023 Aug 25;7:e49738. doi: 10.2196/49738 (PMC10492168; doi:10.2196/49738)
Supplement: Multimedia Appendix 2 [file formative_v7i1e49738_app2.pdf]

Concept 1: A modular CMG device with app

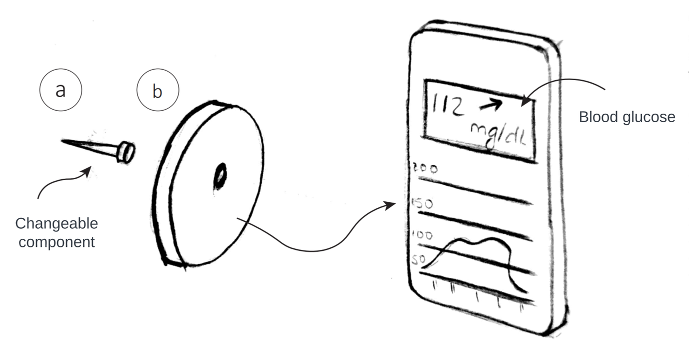

Concept description

Concept 1 is envisioned to be an app combining CGM with contextual information about diabetes related behaviours. The basic idea is to enable broad data collection and easy analysis of trends with CGM data to create insights and self-reflection. Emphasis was put on the CGM device being affordable with electronics and needles being separated.

Summary of suggestions

· Data automatically sent to phone.
· Graphs with arrows (predicting whether BG is going up or down).
· Videos in the app.
· Alarms for highs and lows.
· Immediate use after diagnosis aiming to teach how BG changes over time.
· For persons recently diagnosed with additional occasional use when the illness progresses.
· Lower usage over time with experience.
· Blood glucose and correlation with meals and movement.
· Could such a device be made to work without needles?

Concept 2: Meal app with a focus on blood glucose

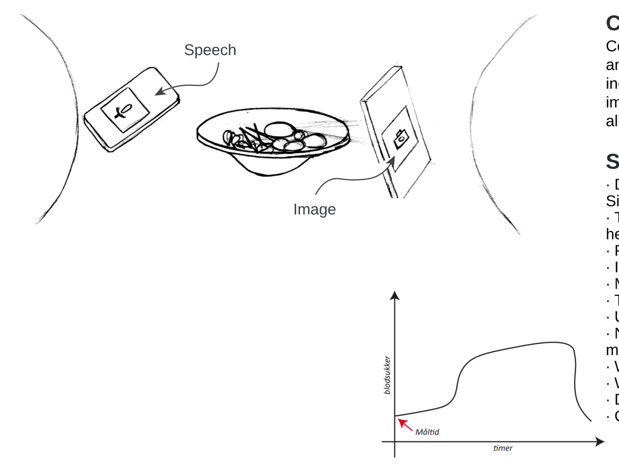

Concept description

Concept 2 is envisioned to be a meal support tool, where meals are documented by photos and can be analysed to displays nutritional information for the meal. Additional features include decision support elements in the form of predicting the blood-glucose based on the image, such that the app provides a best-guess on how the full meal will effect blood glucose allowing the PwD to adjust planned food intake if needed.

Summary of suggestions

· Different ways of adding meals, image, written, using barcodes, or your voice [similar to Siri/Alexa].
· The app should aim to build experience with the aim of not needing to rely on the app for help.
· Preference for taking photos.
· Its not always easy to judge the contents of a meal, app suggestion might help
· Meal advice, recommendations, and BG guidance.
· Timely meal reminders.
· Use colors to specify relevance for BG.
· Needs to teach how the food in the image affects BG i.e., immediate raise, longitude to help manage daily BG.
· Would be nice to in a sense be able to measure long-term BG in relation to meals.
· Would be nice to combine with BG measurements.
· Dexcom/libre style sensors would be nice to use.
· Could be combined with prototype 1.

Concept 3: Home test-kit for diabetes screening

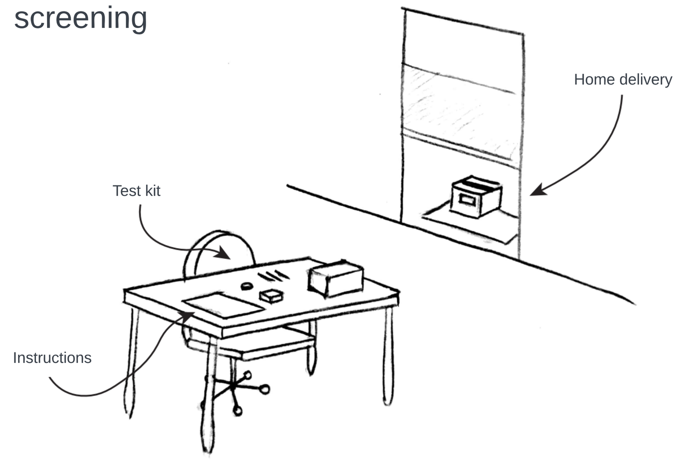

Concept description

The goal of concept 3 is to address issues of late and sometimes coincidental discovery of diabetes and was inspired by other Danish healthcare screening programs. Here, the idea is a home test kit for T2DM, with instructional and motivational material addressing why taking the test is a good idea. The core concept is thus a low-effort, low barrier approach to early discovery of pre-diabetes and undiscovered T2DM.

Summary of suggestions

· What person wouldn't want to know the truth? i.e., the diagnosis.
· More could be done about teaching BG in schools.
· Home test-kit could be points based.
· The kit needs to provide all details.
· The test-kit needs to motivate people to use it and take it seriously.
· Video or textual instructions included.
· Instructions need to educate about general signs of diabetes.
· Needs to motivate not frighten.
· Important that it's possible to follow up on the test-kit with ones own doctor or similar.
· A QR code for more information?
· Could be sent directly to everyone, or high-risk groups i.e., persons with a family history of diabetes.
· Alternatively could be something available at pharmacies.

Prototype 1: Activity-based Continuous Glucose Monitoring (CGM) app

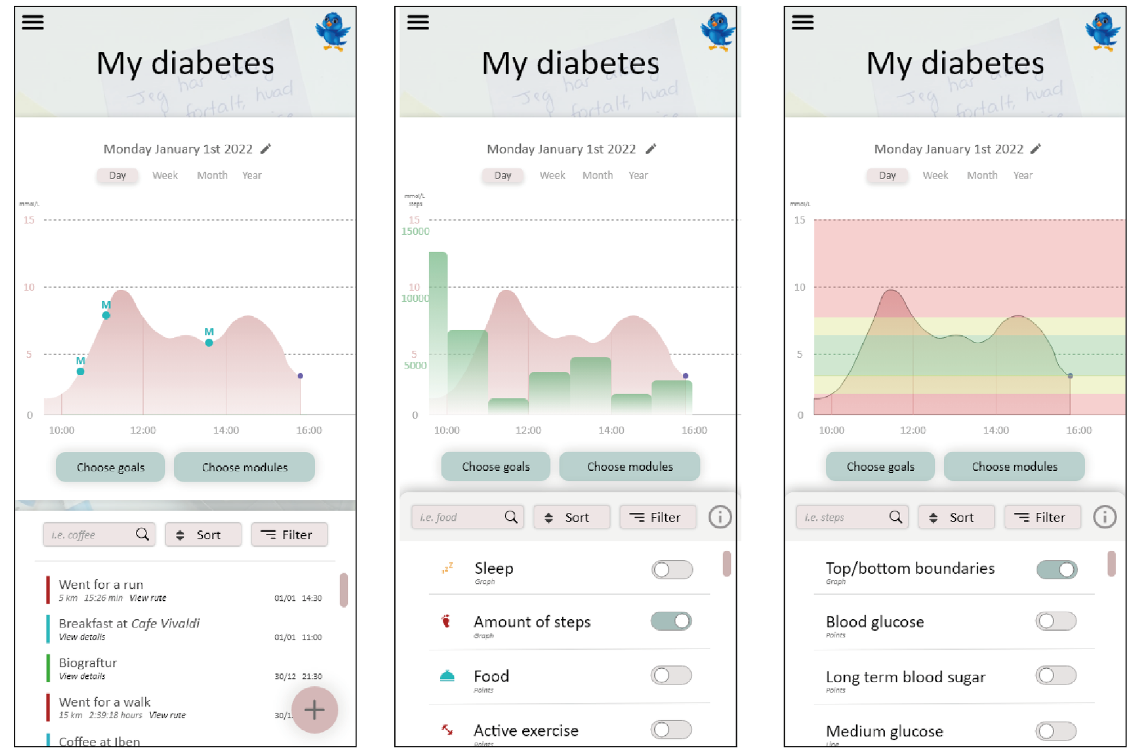

Prototype 2: An online guide to diabetes

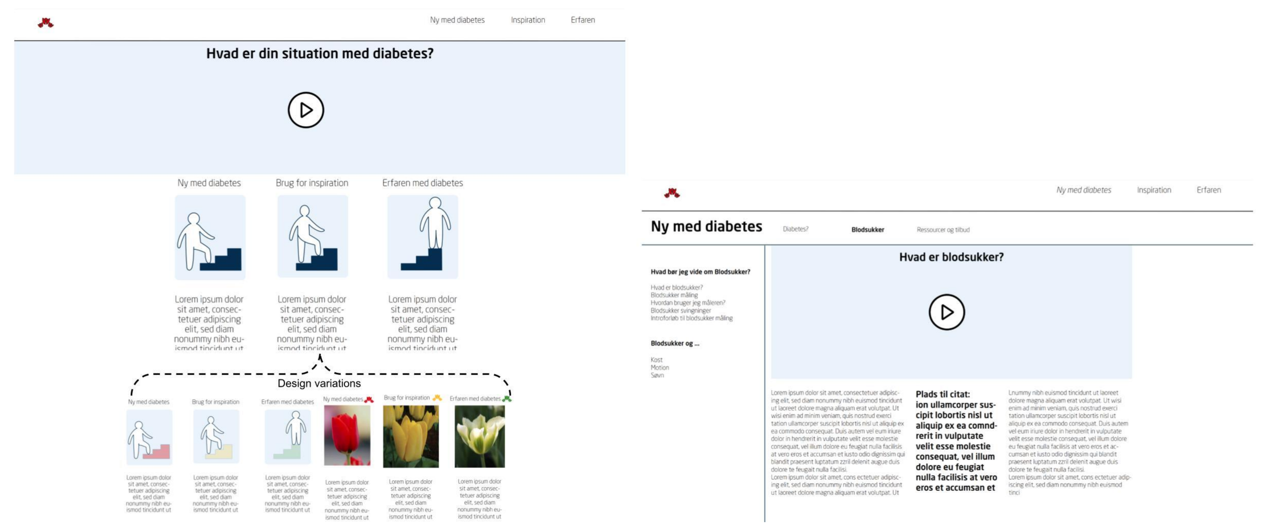

Concept 9: Introduction to diabetes with blood glucose measurements

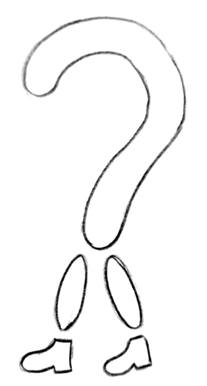

Concept description

Concept 9 is an introduction to diabetes, centred around structured blood glucose measurements, where participants receive an adequate introduction to diabetes with blood glucose measurements to support insights into diabetes and how it impacts the individual.

Summary of suggestions

· An introduction should be standard when diagnosed by one's doctor.
· It should preferably include both physical and digital offers so as many persons as possible can benefit.
· Topics need to include, what diabetes is (too much blood sugar), what damages it can do (comorbidities), what affects blood glucose (mood, exercise, medication) and how you measure/act upon SMBG.
· Video materials, images and descriptions
· Need to know what things are, why and how.
· Categories.
· Should emphasize the importance of measuring BG.
· Should allow for a mix between digital resources and physical ones.
· Chat or hotline where you can ask questions.
· Should be available 24-7.
· The GP should be more thorough with information.
· The introduction should be step by step.
· Should help you learn what parts of my lifestyle should I change.

Concept 8: Diabetes knowledge centre

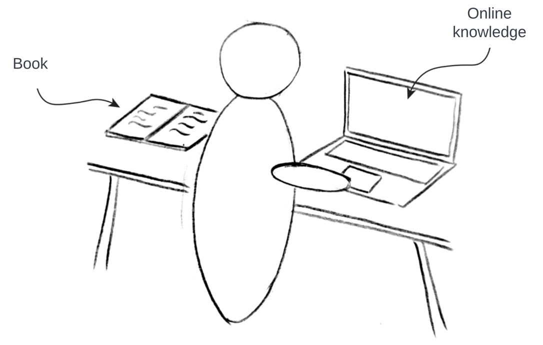

Concept description

Concept 8 is a platform where knowledge and diabetes resources can be gathered with a focus on validated information. Inspiration was drawn from a danish health website "sundhed.dk", but with a focus on aspects of diabetes and the questions that naturally arise from diagnosis and forwards. The platform is curated; knowledge is importantly to be verified by experts and credible sources made available to users to increase transparency and trustworthiness.

Summary of suggestions

· Should have an open-door policy, where you can ask questions almost 24-7.
· Could include small games or quizzes where you test your knowledge.
· Should split content in categories and be an open system.
· The knowledge database should contain extensive knowledge about a variety of diabetes topics – which would make it highly useful.
· Information has to be deep and well documented and preferably aligned with where GPs get information.
· However it also needs to be accessible to the average none-expert.
· It should be structured so its easy to navigate.
· Experts often debate things e.g.., the value of SMBG the knowledge database should address this somehow.

Concept 7: Motivational and discussion groups

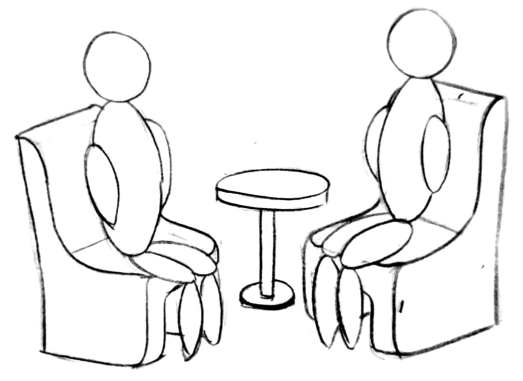

Concept description

Concept 7 is suggested as a possible solution to reduce barriers to joining diabetes groups or finding a mentor, and is envisioned as an index of local groups/persons. The solution aim to ease the process of finding groups with similar lifestyles, needs, or wants based on geographical and other requirements, reducing the effort needed to find like-minded peers.

Summary of suggestions

· Groups need to share interests and hobbies so its possible to know about people before joining.
· The system could facilitate discussions by suggesting topics.
· Focus evenings [workshop like] where persons meet.
· It should be possible to discuss what weighs heavily on a given day.
· Its important that new members are attached to someone with more experience.
· As newly diagnosed its important to receive concrete advice, knowledge and to hear about experiences i.e., with medicine.
· Could be used to find a mentor or make a new friends to discuss diabetes with.
· It is important to consider important questions such as should there be commitments, should it be anonymous.
· Could be facilitated by the DDA.

Concept 6 Individual motivation

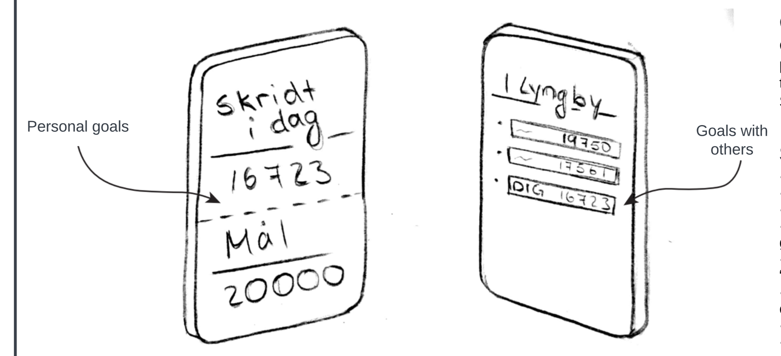

Concept description

Concept 6 is conceptually about finding reasons to engage in physical activity and inspiring people to engage in more physical activity, with the idea to provide inspiration, activities and travel destinations where one can increase physical activity while seeing new places. Statistics, gamification elements, and competitions are among suggested features.

Summary of suggestions

· Group competitions, perhaps by town.
· Inspiration from others in the form of experiences.
· Could motivate exercise, healthy eating, or perhaps structured measurements.
· Perhaps some kind of competition, game or achievement from keeping a stable blood glucose.
· Competitions can also be discouraging so should avoid direct competition i.e., "winning" or "loosing".
· Should preferably include something about BG and diet in addition to exercise so people only have to use a single app.
· Should have rewards for reaching goals.
· "More carrot than stick" – should provide rewards but not force persons.
· Should cheer on persons along the way.
· Could facilitate trying new forms of exercise.
· Could offer help in balancing diet with exercise.

Concept 5 Community motivation

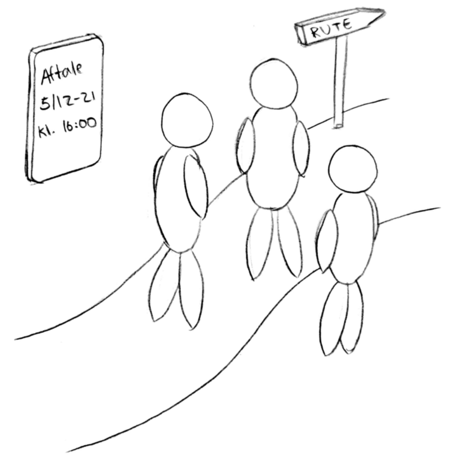

Concept description

Concept 5 is based around motivation and staying motivated in groups by making commitments to the group/community. Based on participant experiences this is envisioned as a hybrid physical/digital solution, supporting both groups based on common activities and when they choose to meet virtually..

Summary of suggestions

· Could/should be a local version of the DDAs "to you that is newly diagnosed with diabetes".
· More workshops in relation to diabetes
· Should be possible to find persons with similar interests, situation and try to match you with similar persons.
· Matching preferred exercise types.
· Add a kind of "moral contract".
· Exercise levels between persons should preferably match.
· If groups don't work, you can easily switch.
· Easy to plan days and times for exercise.
· Could contain other social activities relating to diabetes i.e., cooking.
· Community is important it [exercise] does not have to be related to diabetes.
· Could motivation be facilitated beyond physical boundaries e.g., covid friendly such that motivation can be in person or with others not physically present.

Concept 4: Everday inspiration for living with diabetes

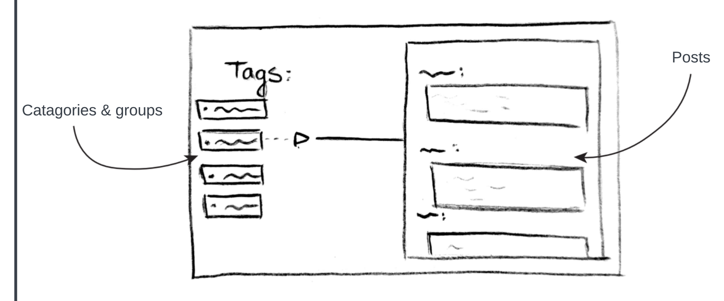

Concept description

Concept 4 relates to searching for, sharing and finding trustworthy inspiration to everyday life with diabetes. Core to the solution is the idea of forum with easily search-able content, to enable broad sharing of knowledge and approaches to everyday life with diabetes. Here PwD can share problems, victories, inspiration, local offers or promote local initiatives and easily locate information relevant to them.

Summary of suggestions

· Top-down categories, diet -> lunch, dinner, carbs etc.
· The forum should be anonymous.
· Anonymity preferred.
· Should have a tone of professionalism.
· Things should not be liked or disliked.
· Should facilitate sharing of experiences.
· Lots of experience can be drawn from existing Facebook diabetes communities.
· The forum should ensure fairness somehow.
· Could perhaps facilitate mentoring or local groups.
· Should be split into easy to find sections like reddit.
· It is important to highlight that inspiration is fine, but it is on each person to take responsibility.
